# Vagal gut-brain signaling mediates amygdaloid plasticity, affect, and pain in a functional dyspepsia model

**DOI:** 10.1172/jci.insight.144046

**Published:** 2021-03-22

**Authors:** Zachary A. Cordner, Qian Li, Liansheng Liu, Kellie L. Tamashiro, Aditi Bhargava, Timothy H. Moran, Pankaj Jay Pasricha

**Affiliations:** 1 Department of Psychiatry and Behavioral Sciences and; 2 Center for Neurogastroenterology, Department of Medicine, Johns Hopkins University School of Medicine, Baltimore, Maryland, USA.; 3 Department of Obstetrics and Gynecology and The Center for Reproductive Sciences, UCSF, San Francisco, California, USA.

**Keywords:** Gastroenterology, Neuroscience, Mast cells, Pain, Psychiatric diseases

## Abstract

Functional dyspepsia (FD) is associated with chronic gastrointestinal distress and with anxiety and depression. Here, we hypothesized that aberrant gastric signals, transmitted by the vagus nerve, may alter key brain regions modulating affective and pain behavior. Using a previously validated rat model of FD characterized by gastric hypersensitivity, depression-like behavior, and anxiety-like behavior, we found that vagal activity — in response to gastric distention — was increased in FD rats. The FD phenotype was associated with gastric mast cell hyperplasia and increased expression of corticotrophin-releasing factor (Crh) and decreased brain-derived neurotrophic factor genes in the central amygdala. Subdiaphragmatic vagotomy reversed these changes and restored affective behavior to that of controls. Vagotomy partially attenuated pain responses to gastric distention, which may be mediated by central reflexes in the periaqueductal gray, as determined by local injection of lidocaine. Ketotifen, a mast cell stabilizer, reduced vagal hypersensitivity, normalized affective behavior, and attenuated gastric hyperalgesia. In conclusion, vagal activity, partially driven by gastric mast cells, induces long-lasting changes in Crh signaling in the amygdala that may be responsible for enhanced pain and enhanced anxiety- and depression-like behaviors. Together, these results support a “bottom-up” pathway involving the gut-brain axis in the pathogenesis of both gastric pain and psychiatric comorbidity in FD.

## Graphical abstract

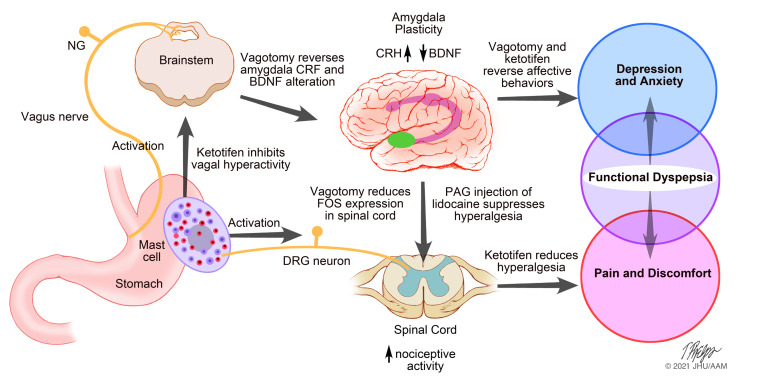

## Introduction

Functional dyspepsia (FD), a highly prevalent clinical gastrointestinal syndrome, has been variously defined but generally includes some form of upper abdominal discomfort or pain. The Rome Committee criteria subclassify FD into 2 types, one of which is defined predominantly by meal-related pain (epigastric pain syndrome; FD/EPS) and the other defined by predominant fullness or early satiety or both (postprandial distress syndrome; FD/PDS), although there are varying degrees of overlap (1, 2). Regardless of the symptomatic phenotype, patients with FD commonly have psychiatric comorbidities, such as anxiety and depression (3, 4). It has been widely accepted that these can drive gastrointestinal symptom perception (“top-down” model; refs. 5, 6). However, recent evidence from animal models (7), as well as human subjects, suggests that gastrointestinal pathology may actually induce anxiety and depression (“bottom-up” model; ref. 8). Thus, the gut-brain axis may actually be involved in a bidirectional role in the genesis of both abdominal symptoms and associated mood disorder (9). However, the mechanisms by which primary gastric insults can initiate changes in affect and mood have not been investigated and are the subject of the present study.

A leading candidate mechanism in mediating these changes is the vagus nerve, a major route of communication between visceral organs and the brain (10). Sensory (afferent) vagal neurons, whose cell bodies are located in the nodose ganglia, project to a variety of visceral organs including the gastrointestinal tract. In the gut, they respond to physiological and pathological stimuli and relay this information to second-order neurons in the nucleus tractus solitarius (NTS). The NTS neurons further project to almost every other brain region, including centers involved in mood regulation such as the amygdala. Indeed, the vagus nerve is known to induce or modulate behavioral changes in response to peripheral inflammatory insults (10) and has been implicated in the pathogenesis of anxiety and depression in irritable bowel syndrome (IBS), contributing directly to affective behavior and indirectly to enhanced nociception in rodent models (11–13).

We therefore hypothesized that increased gastric vagal activity may contribute to the changes in affective behavior and nociception associated with FD, and that gastric mast cells may have a mechanistic role, given their well-established functional relationship with neurons (14). Several studies have reported an increase in mucosal mast cells in the upper gastrointestinal tract in both children and adults with FD, similar to what has been described in patients with IBS (15–17). In the current study, we tested these hypotheses using a previously validated rat model of FD in which mild gastric irritation in neonatal rats induces a state of persistent gastric hyperalgesia, impaired gastric motility, and anxiety-like and depression-like behavior in adulthood, which was associated with brain alterations (7, 18).

## Results

### Vagal activity drives depression- and anxiety-like behaviors in FD rats.

**Figure 1:**
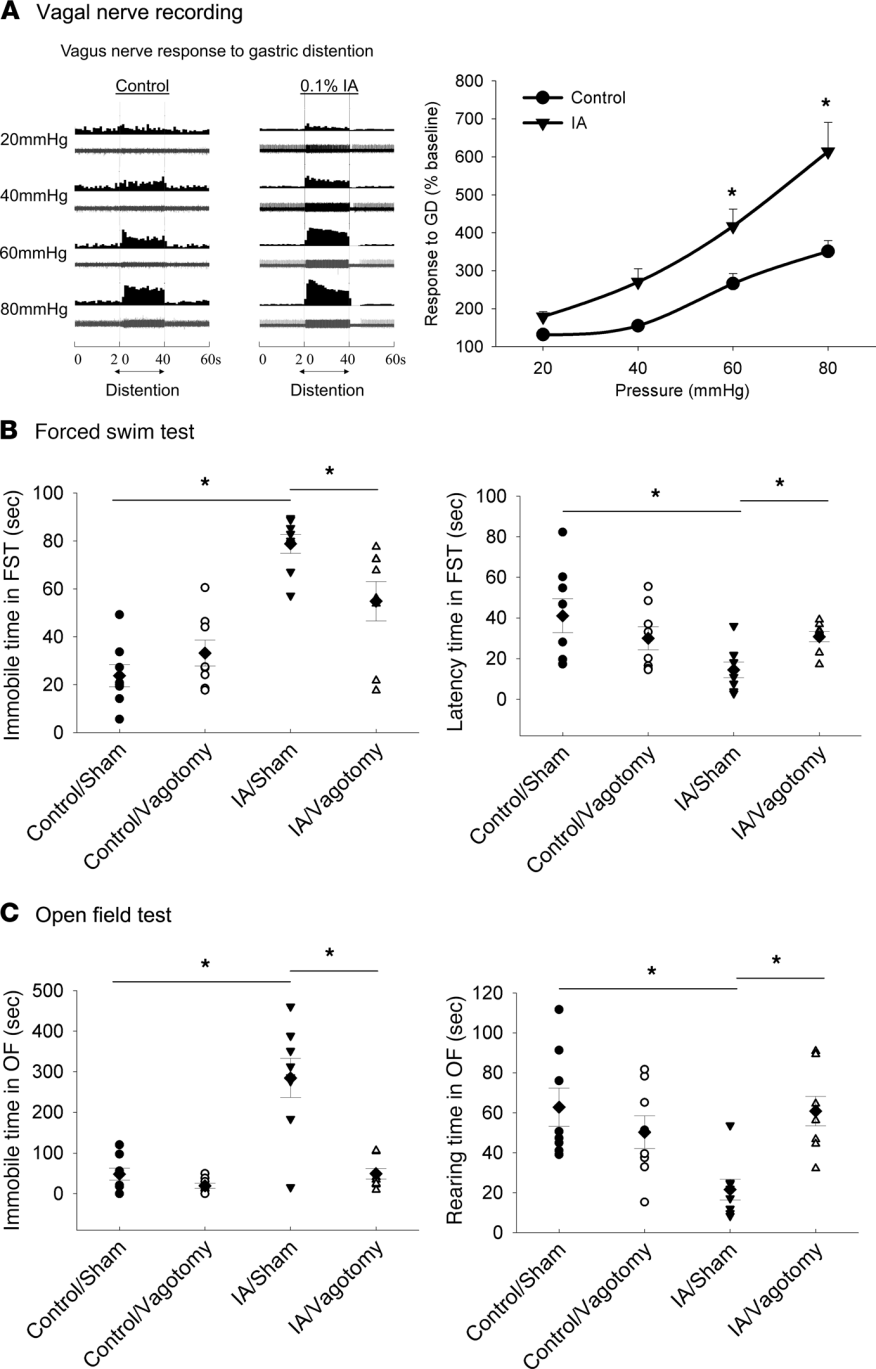
Vagal nerve hypersensitivity to gastric distention and relationship to affect in FD. (**A**) Vagal nerve activity is increased in adult FD rats induced by neonatal intragastric iodoacetamide (IA) as compared with those exposed to vehicle alone (control). The left panel shows an example of single vagal nerve activity in response to gastric distention (GD) (20–40 seconds [s]). The top trace represents the firing frequency (spikes/s) calculated from nerve action potentials as shown in the bottom trace; the right panel shows summated data of vagal nerve activity in response to GD in control and IA rats. Data are expressed as mean ± SEM (*n =* 30–42 fibers from 5–8 rats). Data were analyzed using 2-way ANOVA followed by post hoc testing (*P < 0.05 by Student-Newman-Keuls post hoc test, significantly different from controls at same pressure). (**B** and **C**) Vagotomy reversed depression- and anxiety-like behaviors assessed by forced swim test (**B**) and open field test (**C**). The dot plots represent individual values (circles and triangles) and means (diamonds) ± SEM (bars) (*n =* 8 rats). Data were analyzed using 2-way ANOVA followed by post hoc testing (*P < 0.05 by Student-Newman-Keuls post hoc test, significantly different between 2 groups).

We first examined the vagal afferent responses to gastric distention (GD) in the FD model using single–nerve unit recordings. While baseline activity was similar in both groups, vagal nerve activity in response to GD was significantly increased in FD rats as compared with controls (Figure 1A, 2-way ANOVA; main effect of model, F_1,280_ =23.54, *P <* 0.001; main effect of pressure, F_3,280_ =23.99, *P <* 0.001; interaction of model and pressure, F_3,280_ =2.3, *P =* 0.078). These data indicate that the FD phenotype is associated with gastric vagal hypersensitivity to distention. We next examined the effects of vagotomy on affective behaviors in FD rats.

FD rats that underwent sham surgery showed increased time spent immobile and reduced latency to immobility in the forced swim test (FST), indicative of depression-like behavior. These alterations were reversed by vagotomy (Figure 1B, 2-way ANOVA; immobile time: main effect of model, F_1,28_ = 43.7, *P <* 0.001; main effect of vagotomy, F_1,28_ = 1.54, *P =* 0.224; interaction of model and vagotomy, F_1,28_ = 8.27, *P <* 0.05; latency: main effect of model, F_1,28_ = 5.43, *P <* 0.05; main effect of vagotomy, F_1,28_ = 0.23, *P =* 0.634; interaction of model and vagotomy, F_1,28_ = 6.086, *P <* 0.05).

Furthermore, relative to controls, FD rats showed increased immobile time and decreased rearing time in the open field (OF) test, suggesting an increase in anxiety-like behavior (19, 20) (Figure 1C). These behavioral changes were also normalized by vagotomy (Figure 1C, 2-way ANOVA; immobile: main effect of model, F_1,28_ = 25.9, *P <* 0.001; main effect of vagotomy, F_1,28_ = 25.4, *P <* 0.001; interaction of model and vagotomy, F_1,28_ = 15.59, *P <* 0.001; rearing: main effect of model, F_1,28_ = 3.91, *P <* 0.001; main effect of vagotomy, F_1,28_ = 2.98, *P =* 0.096; interaction of model and vagotomy, F_1,28_ = 11.15, *P <* 0.05). Vagotomy had no effect on these behaviors in control rats.

### FD is associated with gene expression changes in the amygdala that are partially normalized by vagotomy.

**Figure 2:**
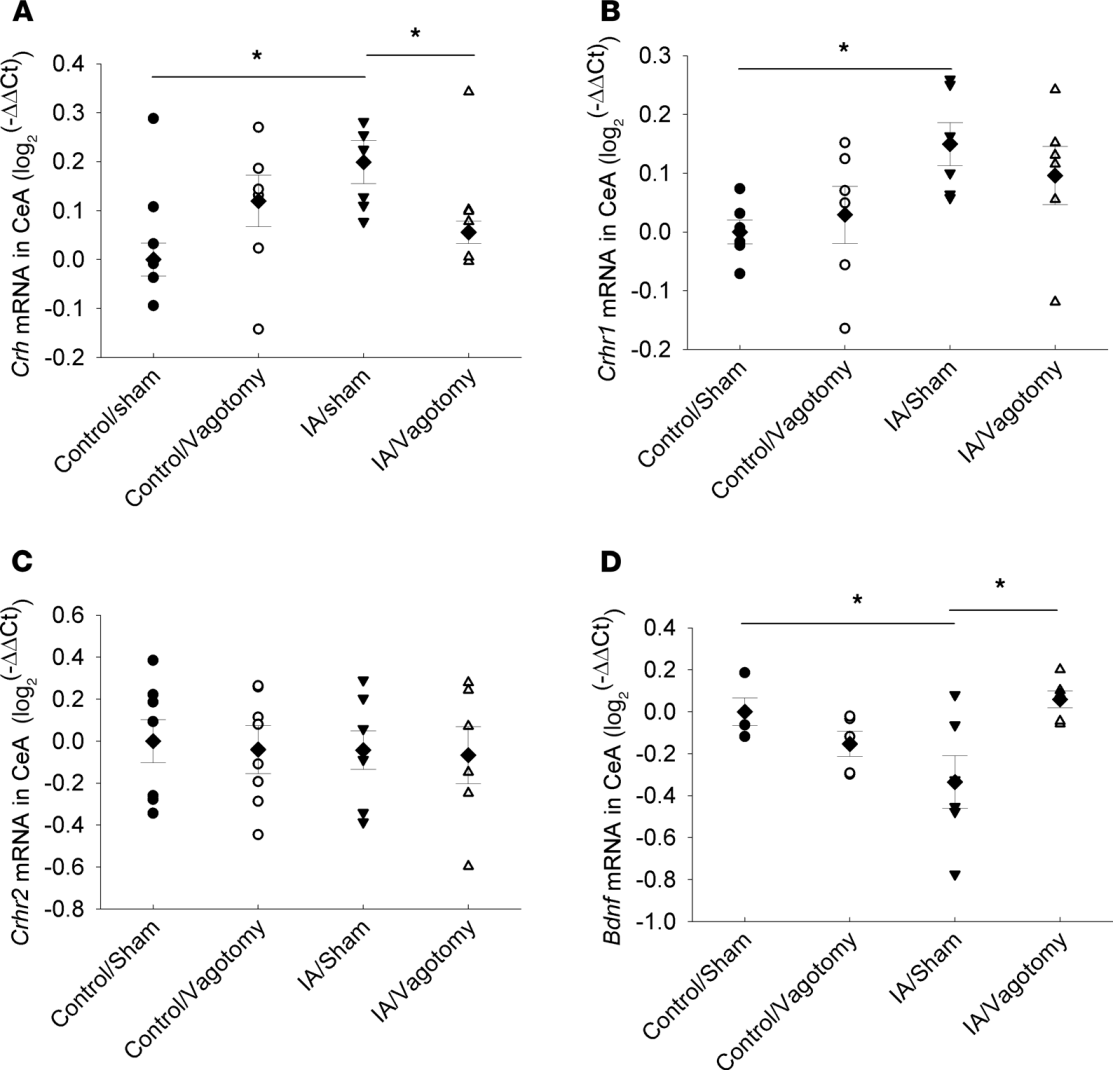
Effects of vagotomy on amygdaloid gene expression in adult FD rats. (**A**–**D**) Expressions of *Crh* (**A**), *Crhr1* (**B**), and *Crhr2* (**C**) receptors and *Bdnf* (**D**) in the central amygdala of control and FD rats were measured by quantitative PCR. The dot plots represent individual values (circles and triangles) with means (diamonds) ± SEM (bars) (*n =* 5–8 rats). Data were analyzed using 2-way ANOVA followed by post hoc testing (*P < 0.05 by Student-Newman-Keuls post hoc test, significantly different between 2 groups).

Given that vagotomy robustly normalized the depressive- and anxiety-like phenotype of FD rats, we further examined the effects of vagotomy on the expression of relevant genes in the amygdala, a key brain nucleus in regulating affective behavior. In the central amygdala (CeA), corticotrophin releasing hormone (*Crh*) gene expression was significantly increased in FD rats, an effect that was reversed by vagotomy (Figure 2A, 2-way ANOVA; main effect of model, F_1,19_ = 2.35, *P =* 0.142; main effect of vagotomy, F_1,19_ = 0.0727, *P =* 0.79; interaction of model and vagotomy, F_1,19_ = 8.882, *P <* 0.05). Furthermore, post hoc tests showed significant differences between control/sham and FD/sham and between FD/sham and FD/vagotomy (Figure 2A). The expression of the *Crh* receptor 1 gene (*Crhr1*; Figure 2B), but not *Crhr2* (Figure 2C), was increased in the CeA of FD rats. However, *Crhr1* expression was not reversed with vagotomy (Figure 2B, 2-way ANOVA; main effect of model, F_1,20_ = 7.089, *P <* 0.05; main effect of vagotomy, F_1,20_ = 0.092, *P =* 0.765; interaction of model and vagotomy, F_1,20_ = 1.039, *P =* 0.32). The expression of the brain-derived neurotrophic factor (*Bdnf*) gene in the CeA was significantly lower in FD rats but normalized by vagotomy (Figure 2D, 2-way ANOVA; main effect of model, F_1,20_ = 0.515, *P =* 0.483; main effect of vagotomy, F_1,20_ = 1.946, *P =* 0.181; interaction of model and vagotomy, F_1,20_ = 10.043, *P <* 0.05). Post hoc tests showed significant differences between control/sham and FD/sham and between FD/sham and FD/vagotomy.

### Vagal activity contributes to nociceptive sensitization via central descending modulatory pathways.

**Figure 3:**
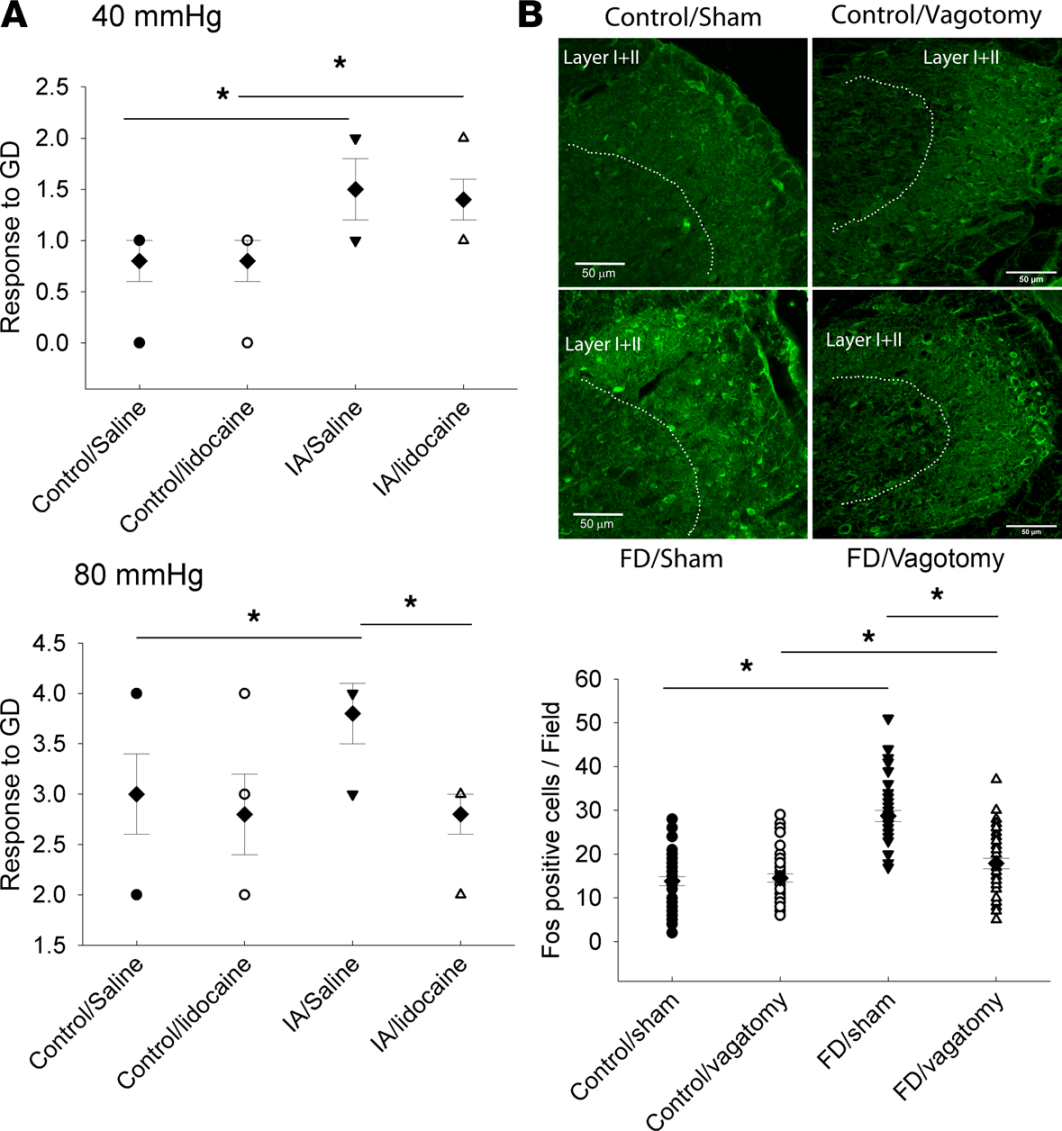
Contribution of central pathways and vagal activity to nociceptive sensitization in adult FD rats induced by neonatal intragastric iodoacetamide (IA) as compared with those exposed to vehicle alone (control). (**A**) Pain behavior in response to GD (40 or 80 mmHg) was measured 10 minutes after lidocaine infusion into the periaqueductal gray (PAG). Increased pain behavioral responses to gastric distention (GD) in FD rats can be attenuated by lidocaine infusion at 80 mmHg, but not 40 mmHg, of pressure. Data were analyzed using 2-way ANOVA followed by post hoc testing. The dot plots represent individual values (circles and triangles) with means (diamonds) ± SEM (bars) (*n =* 5–8 rats) (*P < 0.05 by Student-Newman-Keuls post hoc test, significantly different between 2 groups). (**B**) Vagotomy attenuates increased spinal responsiveness to GD as measured by FOS expression in the dorsal horn of the spinal cord (T8–T10). The top panel shows an example of FOS staining in the layer I and II of the dorsal horn of spinal cord. Scale bar: 50 μm. The bottom panel shows summated data of FOS^+^ cells/field from one side of the dorsal horn. Data are expressed as means ± SEM (*n =* 39–44 field from 4–5 rats/ group). Data were analyzed using 2-way ANOVA followed by post hoc testing. The dot plots represent individual values (circles and triangles) with means (diamonds) ± SEM (bars) (*n =* 39–44 field from 4–5 rats/group) (*P < 0.05 by Student-Newman-Keuls post hoc test, significantly different between 2 groups).

Because the vagus does not directly convey sensory information to the spinal cord, we hypothesized that indirect CNS reflexes, generated in response to vagal signals, may participate in nociception. Both facilitatory and inhibitory pathways from supraspinal centers, such as the ventrolateral periaqueductal gray (PAG) and the rostral ventromedial medulla, can significantly modulate spinal responses to nociceptive signaling from the periphery. We therefore first examined the role of descending pathways in pain behavior in this model by blocking PAG activity with local infusion of lidocaine. As Figure 3A showed, FD rats show increased nociceptive response to GD with both 40 mmHg and 80 mmHg, while PAG infusion with lidocaine attenuated the visceromotor reflex (VMR) response induced by 80 mmHg GD but not 40 mmHg (Figure 3A). Of note, GD with 40 mmHg is not considered noxious in rats (21). These effects began 10 minutes after the infusion (Figure 3A) and began to resolve by 30 minutes after the infusion (data not shown). These results, therefore, indicate that descending pathways from the PAG enhance the spinal responses to noxious stimulation in this model.

We then tested whether increased vagal activity in FD rats contributes to hyperalgesia in these rats. We assessed the effects of vagotomy on expression of FOS (an immediate early gene product that indicates neuronal activation) in layers 1 and 2 of the dorsal horn in thoracic spinal levels 8–10 (T8–T10) in response to GD. We found that GD-induced FOS expression is increased in FD rats, indicating nociceptive sensitization (Figure 3B). Furthermore, vagotomy significantly attenuated, but did not eliminate, this sensitization and had no effect in control rats. Together, these results suggest that vagal hypersensitivity contributes to gastric hyperalgesia in this model, possibly via activating descending facilitatory reflexes from the brain to the spinal cord.

### Increased numbers of mast cells in the stomach may drive vagal hypersensitivity, changes in affect, and hyperalgesia in FD rats.

**Figure 4:**
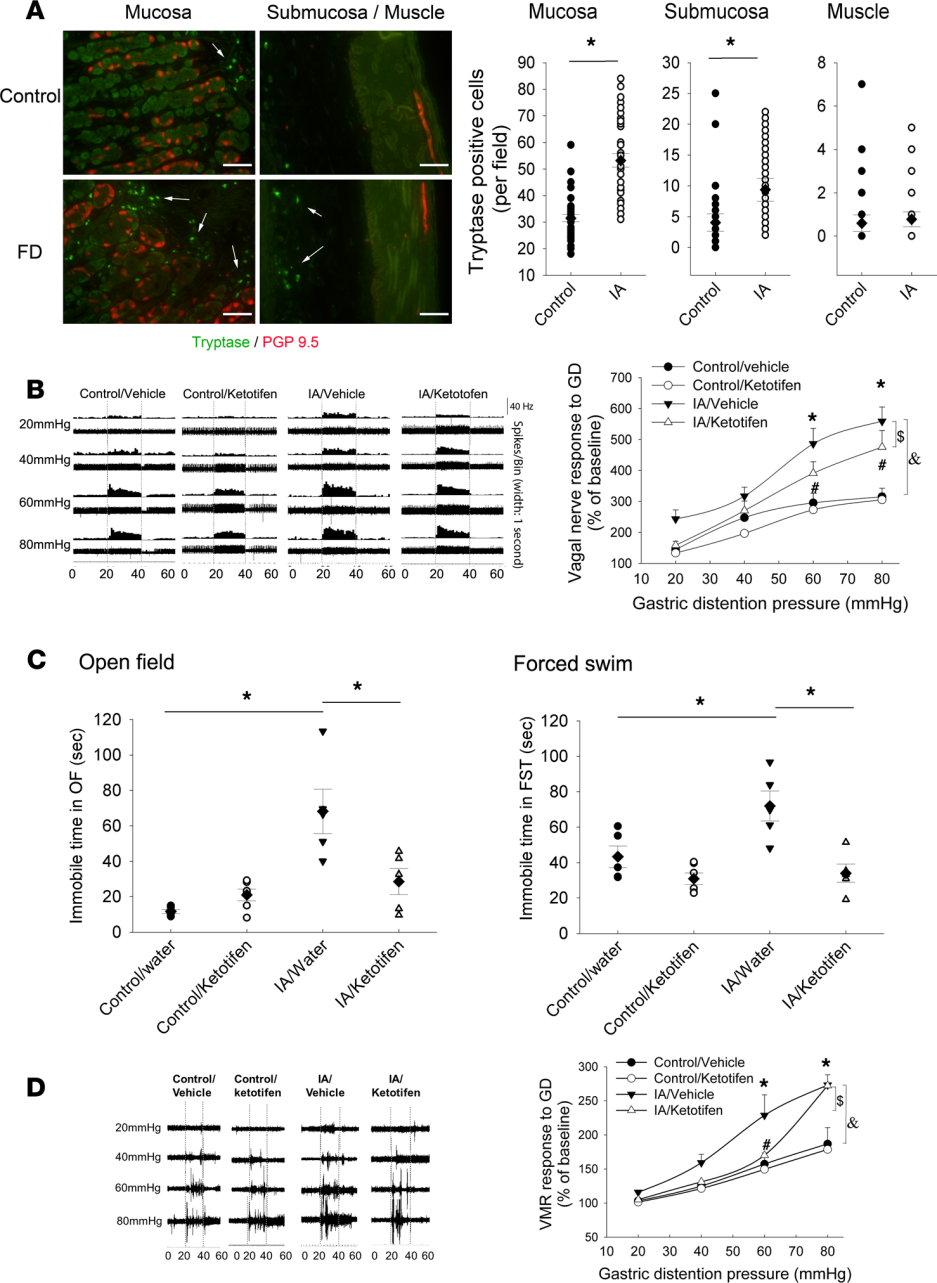
Role of mast cells in vagal hypersensitivity, affect, and pain behavior. (**A**) Gastric mucosa and submucosa stained for mast cell tryptase (green, arrow) and nerves (PGP9.5, red). Scale bar: 15 μm. Summated data, to the right, indicate individual values (circles and triangles) with means (diamonds) ± SEM (bars) (*n =* 40–42 field from 4–5 rats/ group) (*P < 0.05 by Student’s *t* test, significantly different between 2 groups). (**B**) An example of vagal nerve activity in response of gastric distention (GD) (20–40 sec). To the right, summated data on vagal nerve activity expressed as a percentage of the baseline (10–20 s) (means ± SEM; *n =* 24–30 nerves from 5–6 rats/group) and analyzed by 3-way ANOVA and post hoc testing (*P < 0.05, significantly different from control/vehicle group at the indicated GD pressure; ^#^*P <* 0.05, significantly different from IA/vehicle group; ^$^*P <* 0.05, significantly different between IA/vehicle and IA/ketotifen; ^&^*P <* 0.05, significantly different between IA/vehicle and control/vehicle; with Student-Newman-Keuls post hoc test). (**C**) Effects of ketotifen on FD-induced changes on affective behavior, as assessed by open field test (left) and forced swim test (right). Individual values (circles and triangles) with means (diamonds) ± SEM (bars) (*n =* 7–8 rats/group). Data were analyzed using 2-way ANOVA followed by post hoc testing (*P < 0.05 by Student-Newman-Keuls post hoc test, significantly different between 2 groups). (**D**) Effect of ketotifen treatment on hyperalgesia in FD rats in response to gastric distention (GD). An example of visceromotor reflex (VMR) to GD. To the right, summated data (percentage of baseline, means ± SEM; *n =* 7–8 rats/group), analyzed by 2-way ANOVA and post hoc testing (*P < 0.05, significantly different from control/vehicle group at the indicated GD pressure; ^#^*P <* 0.05, significantly different from IA/vehicle group; ^$^*P <* 0.05, significantly different between IA/vehicle and IA/ketotifen; ^&^*P <* 0.05, significantly different between IA/vehicle and control/vehicle; with Student-Newman-Keuls post hoc test).

Having shown that vagal hypersensitivity may maintain the FD phenotype in this model, we next examined the potential mechanistic role of mast cells, based on our original hypothesis and supported by our previously published report (22). We found that tryptase-positive mast cells were significantly increased in the mucosa and submucosa of the stomach of FD rats (Figure 4A, Student’s *t* test: *P <* 0.001 for both mucosa and submucosa). By costaining with PGP9.5, a pan-neuronal marker, it was observed that the mast cells were in close proximity to nerve terminals in the mucosa (Figure 4A). When treated with ketotifen, a histamine H1 receptor antagonist and mast cell stabilizer, we found that vagal hypersensitivity to GD in FD rats was partially reversed (Figure 4B, 3-way ANOVA; main effect of model, F_1,146_ = 66.2, *P <* 0.001; main effect of treatment, F_1,146_ = 11.06, *P <* 0.001; main effect of pressure, F_3,146_ = 51.14, *P <* 0.001).

We then tested the effects of ketotifen on affective behavior in this model. As demonstrated previously, FD rats spent significantly more time immobile in the OF test. This effect was reversed by treatment with ketotifen (Figure 4C, 2-way ANOVA; main effects of model, F_1,17_ = 19.92, *P <* 0.001; main effect of ketotifen treatment, F_1,17_ = 4.45, *P =* 0.05; interaction of model and treatment, F_1,17_ = 11.67, *P <* 0.05). Likewise, FD rats spent significantly more time immobile in the FST, and the effect was reversed by treatment with ketotifen (Figure 4C, 2-way ANOVA; main effects of model, F_1,17_ = 7.34, *P <* 0.05; main effect of ketotifen treatment, F_1,17_ = 18.415, *P <* 0.001; interaction of model and treatment, F_1,17_ = 4.76, *P <* 0.05).

Unlike vagotomy, however, ketotifen administration did not reverse the enhanced *Crh* expression in the CeA in FD rats. *Crh* mRNA was significantly increased in the CeA of FD rats (the log_2_^–ΔΔCt^ in control/water, 0 ± 0.079; control/ketotifen, –0.019 ± 0.072; FD/water, 0.22 ± 0.104; FD/ketotifen, 0.21 ± 0.123). Two-way ANOVA revealed a significant difference between control and FD rats, but it showed no effect of ketotifen (main effect of model, F_1,15_ = 5.623, *P <* 0.05; main effect of ketotifen treatment, F_1,15_ = 0.028, *P =* 0.868; interaction of model and treatment, F_1,15_ = 0.00129, *P =* 0.972).

We also examined the effects of ketotifen on the pain behavior response to GD using the VMR response, measured by electromyography (EMG). As compared with control rats, FD rats showed a significant increase in the VMR response to GD, an effect that was attenuated by ketotifen at lower distention pressures but not at the highest (80 mmHg) (Figure 4D, 2-way ANOVA; main effects of groups, F_3,60_ = 17.1, *P <* 0.001; main effect of pressure, F_3,60_ = 71.5, *P <* 0.001; interaction of groups and pressure, F_9,60_ = 3.6, *P <* 0.001).

These results indicate that activated gastric mast cells play at least a partial role in maintaining hyperactivity of the gastric vagus nerve afferents, affective behavior, and hyperalgesia in this model of FD.

## Discussion

In this study, we hypothesized that the primary manifestations of FD, including both sensory and psychological disturbances, are mediated by aberrant signaling via the abdominal vagus to key regions in the CNS responsible for pain and behavioral responses. Our results (Figure 1) show a robust sensitization of the vagal response to GD, even at pressures previously shown to be in the physiological range as evaluated previously (18, 23). Subdiaphragmatic vagotomy reversed behavioral changes suggestive of depression and anxiety in rats with FD (Figure 1). It is important to acknowledge that this method does have limitations, as do all vagal ablation procedures. The vagus nerve contains approximately 90% afferent and 10% efferent fibers (13), and total vagotomy severs both connections to abdominal viscera, with effects on motility, as well. Though there are other approaches to selectively disturb vagal afferents, they do not seem to inactive all vagal afferents, which presents its own limitations (24). Regardless of the exact mechanisms, however, our results do establish the role of an intact vagus in mediating brain plasticity in the FD model.

We then examined the possible CNS mechanisms responsible for the effect. Ascending fibers in the vagus nerve relay signals to the nucleus tracts solitarius (NTS), which in turn connects to multiple regions of the brain, both directly and indirectly (25–27). Intragastric acid in high concentrations activates neurons in the NTS, lateral parabrachial nucleus, thalamic and hypothalamic paraventricular nucleus, supraoptic nucleus, CeA, and medial/lateral habenula; this effect is nearly abolished by bilateral vagotomy (28). Among these connections, the amygdala stands out as a key component of the limbic system that been implicated in the pathogenesis of anxiety, depression, and other mood disorders. Acute noxious stimulation of the gastrointestinal tract has previously been reported to result in activation and/or plasticity in the amygdala (29–31). Transcriptomic changes in the amygdala have been described in germ-free mice with altered behavioral and nocifensive responses. Functional imaging studies also suggest hyperactivity of the amygdala in humans with IBS (32, 33), indicating a potentially important role of this center in the pathogenesis of chronic gastrointestinal hypersensitivity syndromes that are also associated with altered affective behavior.

We therefore hypothesized that the amygdala may mediate the effects of vagal hypersensitivity on affective behaviors in FD rats, via the production of neuroactive factors. Since we had previously shown that stress-induced circulating adrenocorticotropic hormone (ACTH) and corticosterone levels are increased in this model and that antalarmin, a CRH receptor antagonist, attenuated depression-like behavior (7), we first focused on the CRH system. Our results show increased *Crh* and *Crhr1* (but not *Crhr2*) gene expression in the CeA in FD rats (Figure 2), supporting a potential role for augmented CRH signaling in the pathogenesis of depression and anxiety in this model. Such a role for amygdaloid CRH is consistent with the results of multiple studies in the literature (34–37). We further linked upregulation of the CRH/CRHR1 pathway to vagal activity, since *Crh* expression was normalized after vagotomy, corresponding with normalization of the FD behavioral phenotype. *Crhr1* expression was not significantly changed after vagotomy, suggesting that other, yet unknown, factors may be responsible for this effect.

We also examined the role of BDNF, since its expression is decreased in the amygdala in models of anxiety while enhanced by anxiolytic interventions (38–40). Most antidepressant drugs also cause an increase in BDNF expression (41, 42). Our results show that *Bdnf* gene expression was decreased in rats with FD and normalized after a vagotomy (Figure 2), consistent with its putative role in mood disorders.

Our data therefore indicate that vagal hypersensitivity in a disease model can result in modulation of CRH and BDNF in the amygdala, which, to the best of our knowledge, has not previously been described. However, studies on the effects of vagal nerve stimulation (VNS) provide indirect evidence in support of these results. VNS has been shown to induce plasticity in the amygdala (43) and upregulate CRH in the hypothalamus, as well as increase plasma ACTH and corticosterone levels (44). In healthy mice, VNS also induces BDNF expression and enhances ligand-induced activation of the cognitive receptor for BDNF, TrkB (45, 46). On the other hand, subdiaphragmatic vagotomy decreases *Bdnf* mRNA in the hippocampus (47). Our results indicate a decrease in amygdaloid *Bdnf* gene expression after vagotomy in control rats. In FD rats, however, the effects of vagotomy are the opposite and suggest that, in disease models, increased vagal activity has a pathophysiologic effect on brain plasticity that is different than what the vagus does in health, whether at baseline or in response to VNS.

Together, these findings are in keeping with the results of several other studies. For example, vagotomy consistently blocks depression-like behaviors induced by peripheral injection of LPS and IL-1β–induced behavioral depression (12, 48, 49). In a more recent study, subdiaphragmatic deafferentation of the vagus reduced anxiety-like behavior, as measured by the elevated-plus-maze test, OF test, and food neophobia test (50). On the other hand, data from colonic models demonstrate somewhat conflicting results — with some studies showing that vagotomy blocked the therapeutic effect of probiotics on anxiety- and depression-related behavior in mice under conditions of stress (11, 12), but not in the setting of infectious colitis (51). Thus, it appears that the vagus can both augment and attenuate emotional responses to noxious gastrointestinal stimulation, perhaps depending on the gut region and/or activation of model-specific (e.g., disease versus normal or acute versus chronic) pathways. Our results, therefore, have implications not only for the pathogenesis of affective behavior in FD, but also for possibly predicting the effects of VNS in this condition, which may not be extrapolated from the results obtained in nondisease models or healthy volunteers.

A secondary aim of this study was to examine the role of vagal activity in gastric nociceptive sensitization in FD. Classically, pain is initiated via activation of spinal afferent pathways, and we have previously shown hyperactivity of these nerves in this model of FD (18, 52). It is less certain as to how vagal activity modulates nociception, if at all. An intact vagus and NTS are required for the hyperalgesic effect of proinflammatory cytokines such as IL-1 or TNF (53). Pharmacological or electrical stimulation of the vagus has been reported to result in both facilitation and inhibition of the nociceptive response and appears to involve central circuits, originating in the NTS and then via several relay nuclei, which are activating descending pathways to the spinal cord region to modulate incoming noxious stimuli via spinal afferents (54, 55). In our study, vagotomy did not affect the FOS response to GD in control rats, which is similar to what has been reported earlier by other another group (56). However, vagotomy significantly attenuated, although did not eliminate, enhanced dorsal horn FOS expression in response to GD. Thus, while nociceptive sensitization is still present and maintained by gastric spinal afferents, the subdiaphragmatic vagus is an important contributor. This effect may be mediated by a central pathway, as demonstrated by the reduced pain behavior in response to injection of lidocaine into the ventrolateral PAG. Together, our results support the hypothesis that activated vagal nerves in FD rats attenuate the central inhibitory pathway, resulting in increased sensitivity of the spinal pathway. In this regard, our findings on amygdaloid plasticity suggest that this region may also mediate such a role. The amygdala can receive proalgesic vagal input such as that induced by cholecystokinin (CCK) (13) and, in turn, transmit this to centers of descending pain control, such as the PAG (57, 58). CRH signaling within the CeA has been particularly implicated in the pathogenesis of pathological pain in both somatic and visceral pain states (37, 59–62).

We then examined the peripheral factors that may be responsible for persistent vagal activity, with a focus on gastric mast cells. These cells contain numerous potent effector molecules, which they can release in response to a variety of stimuli and can exert profound effects on secretion, gut barrier integrity, enteric neuronal function, and sensory nerve activity. Their targets include both spinal and vagal nerves; indeed, evidence exists of a bidirectional mast cell–vagus axis (63, 64). We found that mast cell number was increased in the stomach of FD rats and that pharmacological doses of the mast cell stabilizer ketotifen were effective in not only suppressing the vagal hypersensitivity to GD, but also significantly attenuating pain and affective behavior, indicating a potentially key role for these cells in the pathogenesis of FD. This is consistent with our previous report, showing a negative association between mast cell numbers and sucrose intake (22). However, unlike vagotomy, ketotifen did not reverse the increased expression of *Crh* in the amygdala, which may reflect its partial attenuation of vagal hypersensitivity or other mechanisms not related to mast cell activity.

Although the exact mechanism for mast cell proliferation and activation in this model are yet to be fully understood, a variety of experimental models of early life stress suggest that this may be due a disruption of the normal maturing process of the mucosal immune system in neonates (65). Gastric CRH may also play a role in recruiting mast cells in our model of neonatal gastric irritation, as we have previously reported (22). These experimental studies have corroborative evidence in humans. An increase in mast cell density, sometimes along with eosinophils, has also been noted in antral as well as duodenal biopsies from children with FD, and it correlates with symptoms, gastric myoelectrical activity, and altered gastric emptying (15, 16). Gastric mucosal mast cells are also increased in adults with *Helicobacter pylori*–negative FD, particularly in postinfectious cases, where an increased release of histamine and 5-hydroxytryptamine from gastric mucosa has also been noted (66, 67). In a recent study of children with FD, parent report of anxiety and depression correlated significantly with antral mast cell density, but not with any other inflammatory cell type (e.g., eosinophils or T cells) or the presence of esophagitis, gastritis, or duodenitis (68).

**Figure 5:**
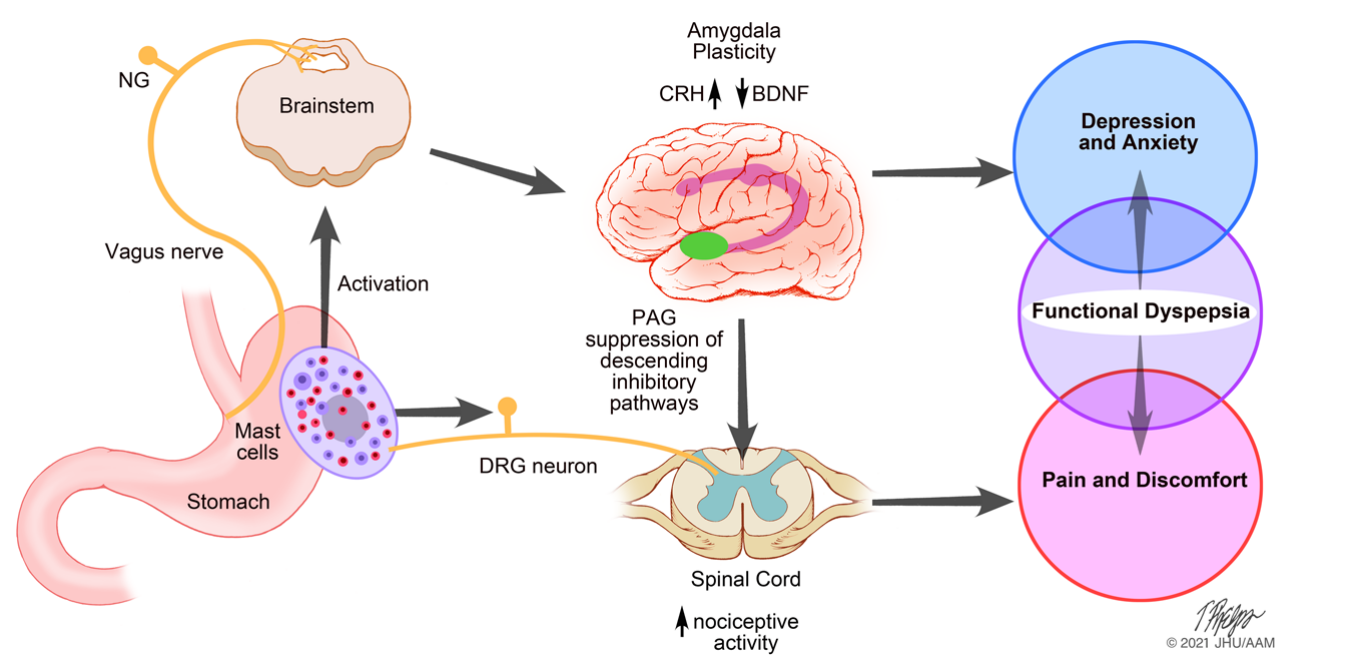
A proposed paradigm for pain and affective behavior in functional dyspepsia. Based on our results, we propose that mast cell hyperplasia and activation results in sensitization of vagal sensory nerves. The latter then causes changes in the amygdaloid region of the brain with associated changes in key neurotransmitters such as CRH (increased) and BDNF (decreased). Furthermore, this is associated with suppression of descending inhibitory pathways that augment nociceptive signals conveyed by gastric spinal sensory nerves. Together, these mechanisms can contribute to pain and discomfort, as well as the psychological morbidities. CRH, corticotrophin releasing hormone; BDNF, brain-derived neurotrophic factor; NG, nodose ganglion; PAG, periaqueductal gray; DRG, dorsal root ganglia.

In conclusion, our study reveals potentially novel biological mechanisms responsible for pain and affective behavior in a model of chronic gastric hypersensitivity/FD. Vagal activity driven in part by gastric mast cells induces long-lasting changes in CRF signaling in the amygdala that may be responsible for both enhanced pain, anxiety, and depression (Figure 5). Together, these results support a role for “bottom-up” pathogenesis of both gastric pain and associated psychological comorbidity, which offers a potentially new paradigm for FD, as well as therapeutic targets based on these aspects of the gut-brain axis.

## Methods

### Animals

Sprague-Dawley rat dams with their litters of pups (10–12 male pups/dam) at P6 were purchased from Harlan Laboratories; female rats were not used because of potentially confounding estrous cycle influences. All rats were housed in a temperature-, humidity-, and light-controlled room (12-hour light/dark cycles). Food and water were available ad libitum. After weaning, rats were housed 2–3 per cage. In all experiments, animals were randomly assigned into the experimental groups.

### FD model

The FD model was generated as previously described (18). Briefly, a weak acid solution, 0.1% iodoacetamide (IA, MilliporeSigma) in 2% sucrose (0.2 mL, Thermo Fisher Scientific), was administered to rat pups (beginning on P10–P12) by oral gavage once a day for 6 days. Control pups received oral gavage of 2% sucrose (0.2 mL). The rats were then weaned at 3 weeks of age and housed under standard conditions until 8–12 weeks of age, when used for the studies.

### Subdiaphragmatic vagotomy

Adult male control and FD rats (12 weeks old) underwent surgery for bilateral subdiaphragmatic vagotomy, as previously described (69, 70). Briefly, after a midline abdominal incision, the esophagus with the associated subdiaphragmatic vagal trunks and their major branches were exposed. The vagal trunks were cut proximal to the bifurcations of the hepatic and celiac branches. Sham surgery was performed in a similar procedure, but the vagus was left intact. Since vagotomy reduces stomach motility, rats were fed liquid food for 3 days before and for 1 week following the surgery. Behavior assessment began 2 weeks after recovery from vagotomy. Depression- and anxiety-like behaviors in FD rats were examined by OF test and FST (see below). Each test was conducted in a day, with at least a 1-day interval between tests. In a separate study, the brains of FD and control rats were collected 3 weeks after vagotomy and were immediately frozen until used for gene expression (See below).

### Spinal FOS response to GD

A balloon was inserted into the stomachs of FD rats during the vagotomy or sham surgery as described above. Two weeks after the surgery, rats were fasted overnight. On the next day, GD with 80 mmHg pressure was conducted for 20 seconds. Twenty minutes later, the rats underwent cardiac perfusion with 0.1M PBS, followed by 300 mL of 4% paraformaldehyde in 0.1M PBS. The T8-T10 spinal cord segments were collected and postfixed with 4% paraformaldehyde overnight and incubated in 30% sucrose solution at 4°C until they sank to the bottom of the vials. Coronal sections (14 μm) of the spinal cord were then used for FOS staining.

### Vagal single–nerve unit recording

Vagal single–nerve unit recording was conducted as previously reported by this laboratory and others, with modifications (18, 71, 72). After separating the vagal nerve from the carotid artery and sympathetic nerve and transecting the vagus below the nodose ganglion, the nerve was teased into fine bundles and split further to obtain a single-unit recording. Nerve activity was recorded by draping the fiber over one arm of the bipolar silver electrode, while an equally thin connective tissue was placed on the other arm of the electrode to allow differential recordings. Single units that innervate to the stomach were identified by consistent spike rate in response to GD. Signals were amplified 1000× with Iso-DAM8A Bio-amplifier (WPI), filtered with 300 Hz to 3 KHz, and monitored with TDS 2012 digital oscilloscope (Tektronix). Then, the wave-mark template in SPIK 2 computer software program (Cambridge Electronic Design) was used to record and analyze nerve activity. GD with graded pressure (20, 40, 60, and 80 mmHg) was applied in an ascending graded manner by rapidly inflating a balloon implanted in the stomach for a duration of 30 seconds using a pressure transducer and sphygmomanometer, with at least a 2-minute interval between stimuli.

### Stereotaxic injection of lidocaine into ventrolateral PAG

A cannula was implanted into the PAG (using stereotaxic coordinates bregma: anterior-posterior [AP], –7.8; lateral [L], 0.5; and dorsal-ventral [DV], –4.5 mm) in 8-week-old FD and control rats (73), while a balloon was implanted for distention and subsequent testing for pain behavior using the abdominal withdrawal reflex (AWR). One week later, after a baseline test (0 minutes) of the AWR response to GD (40 and 80 mmHg), 0.5 μL of 2% lidocaine or normal saline was infused into PAG (bregma: AP, –7.8; L, 0.5; and DV, –5.5 mm) in 80 seconds, and the needle was left for an additional 40 seconds. The AWR response to GD (40 or 80 mmHg, 20 seconds) was measured at 10 minutes after the infusion by behavioral scores as previously described (18). Behavioral responses were graded as: 0, no behavioral response to GD; 1, brief head movement followed by immobility; 2, contraction of abdominal muscles; 3, lifting of abdomen; and 4, body arching, lifting of pelvic structures, and stretching of body. The highest score of behaviors in the 20 seconds of GD period was counted. Behaviors were videotaped and analyzed by a blinded observer.

### Effect of ketotifen on vagal nerve activity and behaviors of IA-treated rats

Ketotifen, a mast cell stabilizer, was purchased from Santa Cruz Biotechnology Inc. (sc-201094). Adult rats that had received neonatal IA or vehicle were treated with ketotifen through drinking water (0.1 mg/mL) for 7 days. The average intake of drinking water by 2 groups of rats was 52–62 mL. There was no significant difference in the amount of drinking water consumed between control and ketotifen-treated rats. On the day after the last treatment, all rats underwent vagal afferent recording in response to gastric distension as described above. In a separate study, rats were treated with ketotifen for 10 days prior to behavioral and gastric nociceptive sensitivity tests using VMR response to GD measured by EMG as we previously reported (18) (see below). Treatment with ketotifen in drinking water was continued during testing.

### Behavioral tests

All behavioral tests are conducted during the light cycle, between 1000 and 1700 hours. Rats were tested in a randomized order.

#### OF.

The OF consists of an arena (60 cm × 60 cm × 40 cm) with a circular inner zone that has a 30 cm diameter. Each rat was gently placed into the center of the field and allowed to explore the arena for 10 minutes. Behavior was scored by a blinded observer for time spent in immobile and rearing.

#### FST.

The FST was conducted using a clear Plexiglas cylinder (65 cm tall × 25 cm diameter) that was filled to 48 cm with 23°C water. On the first day, each rat was placed in the swim chamber and allowed to habituate for 10 minutes. The water was changed, and the swim chamber was cleaned after each rat. Twenty-four hours later, each rat was placed in the swim chamber for 4 minutes. Behavior on the second day was scored by a blinded observer for latency to immobility and total immobility time.

#### VMR.

VMR response to GD was used to assess hyperalgesia in FD rats as we previously described (18). Briefly, a balloon was inserted into the stomach, and a pair of electrodes was implanted into the acromiotrapezius muscle 1 week before the test. The VMR responses to GD were measured by EMG. After basal EMG activity was recorded for 20 seconds, intragastric pressures (20, 40, 60, and 80 mmHg) were applied by a gastric balloon for 20 seconds, and EMG was recorded. EMG activity was calculated as the AUC by the SPIKE2 program. The data were expressed as an increase of GD response to baseline.

### Tissue collection and molecular studies

#### Brain dissection.

Animals were euthanized during the light cycle between 1000 and 1400 hours. After rapid decapitation, rat brains were flash frozen in dry ice–cold 2-methybutane and then placed in powdered dry ice for at least 10 minutes. The brains were stored at –80°C until use. To dissect the brain regions, 300 μm coronal brain slices were sectioned at the level of amygdala. CeA (bregma: –1.92 mm to –2.76 mm) were dissected (73). The tissue was stored at –80°C until use for quantitative PCR (qPCR).

#### qPCR.

Total RNA was extracted from the CeA using RNeasy plus micro kits (Qiagen), and 100 ng RNA was used to make cDNA using SuperScript III Reverse Transcriptase (Thermo Fisher Scientific). Taqman assays for specific genes of interest were used to examine the expression of the genes for *Crh* (Taqman assay ID: Rn01462137_m1), *Crhr1* (Taqman assay ID: Rn00578611_m1), *Crhr2* (Taqman assay ID: Rn00575617_m1), and *Bdnf* (Taqman assay ID: Rn02531967_s1) (Thermo Fisher Scientific). The qPCR was normalized by house-keeping gene, Hprt1 (Taqman assay ID: Rn01527840_m1). qPCR was performed for each sample in triplicate using TaqPath qPCR Master Mix (Thermo Fisher Scientific) following manufacturer’s suggested protocol. To calculate the gene expression, a threshold was set in the linear range of amplification plot in the Rotor-gene Q software. The mean of threshold cycle number (Ct) from the triplicate of target gene was subtracted to that of Hprt1 (ΔCt). The mean of ΔCt from control/sham group, as reference, was subtracted by each ΔCt of target genes, resulting in ΔΔCt. The gene expression was presented as log_2_^–ΔΔCt^ relative to control/sham group.

#### IHC for tryptase and FOS.

For tryptase staining in the stomach, paraffin-embedded tissue sections were used. After deparaffinizing, sections were washed with PBS for 5 minutes, 3 times, followed by blocking with 10% normal goat serum (NGS) and 5% BSA in PBS in 1 hour. The sections were then incubated overnight with mouse anti–mast cell tryptase (1:300, NBP2-26444, Novus Biologicals) and rabbit anti-PGP9.5 (1:400, Z5116 DAKO/Aligent) in PBS containing 5% NGS, 1 % BSA, and 0.1% Triton X-100. After 3 washes with PBS, sections were incubated with goat anti–mouse IgG conjugated with Alexa 488 and goat anti–rabbit IgG conjugated with Alexa 594 (1:500, A-11001 and A-11012, respectively; Thermo Fisher Scientific) for 2 hours. After the wash, the slides were mounted with ProLong Gold Antifade Mountant solution (P36931, Thermo Fisher Scientific).

For FOS staining, the spinal cord (T8–T10) was collected and sectioned to 14 μm slices that were mounted on slides. The immunostaining procedure was similar to that for tryptase, except goat anti-FOS (sc271243, Santa Cruz Biotechnology Inc.) at 1:100 dilution was used.

The images were captured by a fluorescent microscope with 20× lens. The images were analyzed using ImageJ (NIH) software. Four to 6 sections from each animal and 4–5 rats in each group were analyzed.

### Statistics

Data were analyzed by 2-way ANOVA or 2-tailed Student’s *t* test using SigmaPlot (Systat software Inc.), unless otherwise specified. If a significant difference was detected, a Student-Newman-Keuls post hoc test was used to evaluate differences between individual groups. Data are expressed as mean ± SEM of the group (*n =* 6–8, unless otherwise noted). For all tests, *P <* 0.05 was considered significant.

### Study approval

All procedures were conducted in accordance with the *Guide for the Care and Use of Laboratory Animals* (National Academies Press, 2011) and were approved by the IACUC of the Johns Hopkins University.

## Author contributions

Conceptualization was contributed by PJP, THM, and AB. Formal analysis was contributed by QL. Funding acquisition was contributed by PJP and AB. Investigation was contributed by ZAC, LL, KLT, and QL. Methodology was contributed by THM, QL, KLT, and PJP. Project administration was contributed by PJP and QL. Resources were contributed by PJP and THM. Supervision was contributed by PJP and THM. Writing of the original draft was contributed by PJP and QL. All authors reviewed and edited the manuscript.

## Version 1 02/16/2021

In-Press Preview

## Version 2 03/22/2021

Electronic publication

## References

[1] Aziz I (2018). Epidemiology, clinical characteristics, and associations for symptom-based Rome IV functional dyspepsia in adults in the USA, Canada, and the UK: a cross-sectional population-based study. Lancet Gastroenterol Hepatol.

[2] Drossman DA. Functional Gastrointestinal Disorders: History, Pathophysiology, Clinical Features and Rome IV [published online February 19, 2016]. Gastroenterology. 10.1053/j.gastro.2016.02.032 27144617

[3] Aro P (2009). Anxiety is associated with uninvestigated and functional dyspepsia (Rome III criteria) in a Swedish population-based study. Gastroenterology.

[4] Mak AD (2012). Dyspepsia is strongly associated with major depression and generalised anxiety disorder - a community study. Aliment Pharmacol Ther.

[5] Geeraerts B, Tack J (2008). Functional dyspepsia: past, present, and future. J Gastroenterol.

[6] Geeraerts B (2008). Influence of experimentally induced anxiety on rectal sensorimotor function in healthy humans. Neurogastroenterol Motil.

[7] Liu L (2011). Transient gastric irritation in the neonatal rats leads to changes in hypothalamic CRF expression, depression- and anxiety-like behavior as adults. PLoS One.

[8] Koloski NA (2012). The brain—gut pathway in functional gastrointestinal disorders is bidirectional: a 12-year prospective population-based study. Gut.

[9] Greenwood-Van Meerveld B, Johnson AC (2017). Stress-induced chronic visceral pain of gastrointestinal origin. Front Syst Neurosci.

[10] Breit S (2018). Vagus nerve as modulator of the brain-gut axis in psychiatric and inflammatory disorders. Front Psychiatry.

[11] Bravo JA (2011). Ingestion of Lactobacillus strain regulates emotional behavior and central GABA receptor expression in a mouse via the vagus nerve. Proc Natl Acad Sci U S A.

[12] Konsman JP (2000). The vagus nerve mediates behavioural depression, but not fever, in response to peripheral immune signals; a functional anatomical analysis. Eur J Neurosci.

[13] Wang EM (2015). Vagal afferent-dependent cholecystokinin modulation of visceral pain requires central amygdala NMDA-NR2B receptors in rats. Neurogastroenterol Motil.

[14] Forsythe P (2019). Mast cells in neuroimmune interactions. Trends Neurosci.

[15] Schappi MG (2008). Mast cell-nerve interactions in children with functional dyspepsia. J Pediatr Gastroenterol Nutr.

[16] Friesen CA (2008). Antral inflammatory cells, gastric emptying, and electrogastrography in pediatric functional dyspepsia. Dig Dis Sci.

[17] Friesen C (2020). A cross-sectional study of nausea in functional abdominal pain: relation to mucosal mast cells and psychological functioning. BMC Gastroenterol.

[18] Liu LS (2008). A rat model of chronic gastric sensorimotor dysfunction resulting from transient neonatal gastric irritation. Gastroenterology.

[19] Ramos A, Mormede P (1998). Stress and emotionality: a multidimensional and genetic approach. Neurosci Biobehav Rev.

[20] Sturman O (2018). Exploratory rearing: a context- and stress-sensitive behavior recorded in the open-field test. Stress.

[21] Sakurai J (2008). Activation of extracellular signal-regulated protein kinase in sensory neurons after noxious gastric distention and its involvement in acute visceral pain in rats. Gastroenterology.

[22] Hagiwara SI (2018). Gastric corticotropin-releasing factor influences mast cell infiltration in a rat model of functional dyspepsia. PLoS One.

[23] Ozaki N (2002). Models of gastric hyperalgesia in the rat. Am J Physiol Gastrointest Liver Physiol.

[24] Wang YB (2020). Dissecting the role of subtypes of gastrointestinal vagal afferents. Front Physiol.

[25] Ricardo JA, Koh ET (1978). Anatomical evidence of direct projections from the nucleus of the solitary tract to the hypothalamus, amygdala, and other forebrain structures in the rat. Brain Res.

[26] van der Kooy D (1984). The organization of projections from the cortex, amygdala, and hypothalamus to the nucleus of the solitary tract in rat. J Comp Neurol.

[27] Zardetto-Smith AM, Gray TS (1990). Organization of peptidergic and catecholaminergic efferents from the nucleus of the solitary tract to the rat amygdala. Brain Res Bull.

[28] Michl T (2001). Vagal afferent signaling of a gastric mucosal acid insult to medullary, pontine, thalamic, hypothalamic and limbic, but not cortical, nuclei of the rat brain. Pain.

[29] Nakagawa T (2003). Differential patterns of c-fos mRNA expression in the amygdaloid nuclei induced by chemical somatic and visceral noxious stimuli in rats. Neurosci Lett.

[30] Suwanprathes P (2003). c-Fos immunoreactivity in the brain after esophageal acid stimulation. Am J Med.

[31] Greenwood-Van Meerveld B (2006). Long-term expression of corticotropin-releasing factor (CRF) in the paraventricular nucleus of the hypothalamus in response to an acute colonic inflammation. Brain Res.

[32] Hong JY (2016). Altered brain responses in subjects with irritable bowel syndrome during cued and uncued pain expectation. Neurogastroenterol Motil.

[33] Keita AV, Soderholm JD (2010). The intestinal barrier and its regulation by neuroimmune factors. Neurogastroenterol Motil.

[34] Veinante P (2013). The amygdala between sensation and affect: a role in pain. J Mol Psychiatry.

[35] Shekhar A (2005). Role of stress, corticotrophin releasing factor (CRF) and amygdala plasticity in chronic anxiety. Stress.

[36] Tache Y (2009). A role for corticotropin-releasing factor in functional gastrointestinal disorders. Curr Gastroenterol Rep.

[37] Tache Y (2018). Brain and gut CRF signaling: biological actions and role in the gastrointestinal tract. Curr Mol Pharmacol.

[38] Smith JP (2016). Intensity of anxiety is modified via complex integrative stress circuitries. Psychoneuroendocrinology.

[39] Sagarkar S (2017). Minimal traumatic brain injury causes persistent changes in DNA methylation at BDNF gene promoters in rat amygdala: a possible role in anxiety-like behaviors. Neurobiol Dis.

[40] You C (2014). Reversal of deficits in dendritic spines, BDNF and Arc expression in the amygdala during alcohol dependence by HDAC inhibitor treatment. Int J Neuropsychopharmacol.

[41] Castren E, Kojima M (2017). Brain-derived neurotrophic factor in mood disorders and antidepressant treatments. Neurobiol Dis.

[42] Duman RS (2021). Role of BDNF in the pathophysiology and treatment of depression: activity-dependent effects distinguish rapid-acting antidepressants. Eur J Neurosci.

[43] Hachem LD (2018). The vagus afferent network: emerging role in translational connectomics. Neurosurg Focus.

[44] Hosoi T (2000). Electrical stimulation of afferent vagus nerve induces IL-1beta expression in the brain and activates HPA axis. Am J Physiol Regul Integr Comp Physiol.

[45] Furmaga H (2012). Vagal nerve stimulation rapidly activates brain-derived neurotrophic factor receptor TrkB in rat brain. PLoS One.

[46] Follesa P (2007). Vagus nerve stimulation increases norepinephrine concentration and the gene expression of BDNF and bFGF in the rat brain. Brain Res.

[47] O’Leary OF (2018). The vagus nerve modulates BDNF expression and neurogenesis in the hippocampus. Eur Neuropsychopharmacol.

[48] Bluthe RM (2000). Role of interleukin-1beta and tumour necrosis factor-alpha in lipopolysaccharide-induced sickness behaviour: a study with interleukin-1 type I receptor-deficient mice. Eur J Neurosci.

[49] Bluthe RM (1994). Lipopolysaccharide induces sickness behaviour in rats by a vagal mediated mechanism. C R Acad Sci III.

[50] Klarer M (2014). Gut vagal afferents differentially modulate innate anxiety and learned fear. J Neurosci.

[51] Bercik P (2010). Chronic gastrointestinal inflammation induces anxiety-like behavior and alters central nervous system biochemistry in mice. Gastroenterology.

[52] Liu LS (2011). The analgesic effects of the GABAB receptor agonist, baclofen, in a rodent model of functional dyspepsia. Neurogastroenterol Motil.

[53] Watkins LR, Maier SF (2000). The pain of being sick: implications of immune-to-brain communication for understanding pain. Annu Rev Psychol.

[54] Randich A, Gebhart GF (1992). Vagal afferent modulation of nociception. Brain Res Brain Res Rev.

[55] Watkins LR (1994). Neurocircuitry of illness-induced hyperalgesia. Brain Res.

[56] Traub RJ (1996). Differential c-fos expression in the nucleus of the solitary tract and spinal cord following noxious gastric distention in the rat. Neuroscience.

[57] Pavlovic ZW, Bodnar RJ (1998). Opioid supraspinal analgesic synergy between the amygdala and periaqueductal gray in rats. Brain Res.

[58] Li JN, Sheets PL (2018). The central amygdala to periaqueductal gray pathway comprises intrinsically distinct neurons differentially affected in a model of inflammatory pain. J Physiol.

[59] Tache Y (2015). Corticotrophin-releasing factor 1 activation in the centralamygdale and visceral hyperalgesia. Neurogastroenterol Motil.

[60] Su J (2015). Injection of corticotropin-releasing hormone into the amygdala aggravates visceral nociception and induces noradrenaline release in rats. Neurogastroenterol Motil.

[61] Lennon VA (2003). Immunization with neuronal nicotinic acetylcholine receptor induces neurological autoimmune disease. J Clin Invest.

[62] Johnson AC (2015). Knockdown of corticotropin-releasing factor in the central amygdala reverses persistent viscerosomatic hyperalgesia. Transl Psychiatry.

[63] Gottwald T (1997). Effect of truncal vagotomy and capsaicin on mast cells and IgA-positive plasma cells in rat jejunal mucosa. Neurogastroenterol Motil.

[64] Stead RH (2006). Vagal influences over mast cells. Auton Neurosci.

[65] Pohl CS (2015). Early-life stress origins of gastrointestinal disease: animal models, intestinal pathophysiology, and translational implications. Am J Physiol Gastrointest Liver Physiol.

[66] Li X (2010). The study on the role of inflammatory cells and mediators in post-infectious functional dyspepsia. Scand J Gastroenterol.

[67] Hall W (2003). Gastric mucosal mast cells are increased in Helicobacter pylori-negative functional dyspepsia. Clin Gastroenterol Hepatol.

[68] Schurman JV (2010). Symptoms and subtypes in pediatric functional dyspepsia: relation to mucosal inflammation and psychological functioning. J Pediatr Gastroenterol Nutr.

[69] Moran TH (1997). Vagal afferent and efferent contributions to the inhibition of food intake by cholecystokinin. Am J Physiol.

[70] Smedh U, Moran TH (2006). The dorsal vagal complex as a site for cocaine- and amphetamine-regulated transcript peptide to suppress gastric emptying. Am J Physiol Regul Integr Comp Physiol.

[71] Kang YM (2004). Sensitization of mechanosensitive gastric vagal afferent fibers in the rat by thermal and chemical stimuli and gastric ulcers. J Neurophysiol.

[72] Sengupta JN (2004). Response properties of antral mechanosensitive afferent fibers and effects of ionotropic glutamate receptor antagonists. Neuroscience.

[73] Paxinos G, Watson C. *The Rat Brain in Stereotaxic Coordinates*. Academic Press; 2007.

